# Human plague associated with Tibetan sheep originates in marmots

**DOI:** 10.1371/journal.pntd.0006635

**Published:** 2018-08-16

**Authors:** Ruixia Dai, Baiqing Wei, Haoming Xiong, Xiaoyan Yang, Yao Peng, Jian He, Juan Jin, Yumeng Wang, Xi Zha, Zhikai Zhang, Ying Liang, Qingwen Zhang, Jianguo Xu, Zuyun Wang, Wei Li

**Affiliations:** 1 Qinghai Institute for Endemic Disease Control and Prevention, Xining, China; 2 National Institute for Communicable Disease Control and Prevention, China CDC, Changping, Beijing, China; 3 State Key Laboratory of Infectious Disease Prevention and Control, Beijing, China; 4 Collaborative Innovation Center for Diagnosis and Treatment of Infectious Disease, Hangzhou, China; 5 Center for Disease Control and Prevention of Tibet Autonomous Region, Lhasa, China; Faculté de Médecine,Aix-Marseille Université, FRANCE

## Abstract

The Qinghai-Tibet plateau is a natural plague focus and is the largest such focus in China. In this area, while *Marmota himalayana* is the primary host, a total of 18 human plague outbreaks associated with Tibetan sheep (78 cases with 47 deaths) have been reported on the Qinghai-Tibet plateau since 1956. All of the index infectious cases had an exposure history of slaughtering or skinning diseased or dead Tibetan sheep. In this study, we sequenced and compared 38 strains of *Yersinia pestis* isolated from different hosts, including humans, Tibetan sheep, and *M*. *himalayana*. Phylogenetic relationships were reconstructed based on genome-wide single-nucleotide polymorphisms identified from our isolates and reference strains. The phylogenetic relationships illustrated in our study, together with the finding that the Tibetan sheep plague clearly lagged behind the *M*. *himalayana* plague, and a previous study that identified the Tibetan sheep as a plague reservoir with high susceptibility and moderate sensitivity, indicated that the human plague was transmitted from Tibetan sheep, while the Tibetan sheep plague originated from marmots. Tibetan sheep may encounter this infection by contact with dead rodents or through being bitten by fleas originating from *M*. *himalayana* during local epizootics.

## Author summary

Plague is mainly a disease of wild rodents, and their parasitic fleas are considered the transmitting vectors. However, human plague originating from *Ovis aries* (Tibetan sheep) is found in the Qinghai-Tibet plateau in China, where *Marmota*. *himalayana* is the primary plague host. Tibetan sheep-related human plague infection is always associated with slaughtering or skinning diseased or dead Tibetan sheep. The plague in Tibetan sheep clearly lags that in *M*. *himalayana*. In this study, we performed a genome-wide single nucleotide polymorphism analysis of Tibetan sheep-related plague events, including pathogens isolated from humans, Tibetan sheep, and marmots. Through genomic analysis, together with the epidemiological connections, we confirmed that human plague came from Tibetan sheep, and the Tibetan sheep plague originated from marmots. Tibetan sheep account for about 1/3 of the total number of sheep in China. Tibetan sheep and goats are important domestic livestock on the Qinghai-Tibet plateau. Therefore, the hazards of Tibetan sheep plague should not be underestimated.

## Introduction

Plague is an acute infectious disease caused by *Yersinia pestis* that killed millions of people in Europe in the 14th century and tens of thousands in China in the 19^th^ century [1]. Plague is mainly a disease of wild rodents, and their parasitic fleas are considered the transmitting vectors. So far, four subspecies of *Y*. *pestis* have been recognized on the basis of their biochemical properties: *Y*. *pestis* antiqua, mediaevalis, orientalis, and pestoides (microtus) [2,3]. To date, at least 12 plague foci covering >1.4 million km^2^ have been identified in China [4]; the largest focus is the *Marmota himalayana* focus on the Qinghai-Tibet plateau in northwestern China. The overwhelming majority of *Y*. *pestis* pathogens on the Qinghai-Tibet plateau are biovar antiqua, with the exception of biovar microtus (qinghaiensis) in the *Microtus fuscus* focus, which is located in Chengduo county in Qinghai Province and in Shiqu county in Sichuan Province [4].

The Qinghai-Tibet plateau is the highest risk area for human plague in China and *M*. *himalayana* is the primary host in this area. The pathogen *Y*. *pestis* (biovar antiqua) in the Qinghai-Tibet plateau *M*. *himalayana* natural plague focus frequently causes pneumonic and septicemic plague with high mortality. Other rodents (*Allactaga sibirica*, *Mus musculus*, *Cricetulus migratorius*, *Microtus oeconomus*, and *Ochotona daurica*), some wild animals (foxes, lynxes, and badgers), and domestic animals (sheep, cats, and dogs) have been found to be infected by *Y*. *pestis* [5]. Human plague originating from *Ovis aries* (Tibetan sheep) was first reported in 1956 in Qinghai Province [5], though no bacterial evidence was obtained at that time. Tibetan sheep account for ~1/3 of the total number of sheep in China [6]. And the distribution areas of Tibetan sheep plague broadly overlap with the habitat of marmots in the Qinghai-Tibet plateau *M*. *himalayana* plague focus [6,7]. In August 1975, a patient suffered from plague after butchering a dead Tibetan sheep in Yushu Prefecture, Qinghai Province. The meat of the sheep was eaten by 10 people; two individuals suffered intestinal plague that then developed into pneumonic plague, and one died [5]. Three *Y*. *pestis* strains were isolated from the dead individual, Tibetan sheep, and *Capra aegagrus hircus* (Tibetan goat). This incident was the first time that human plague associated with Tibetan sheep or Tibetan goats was confirmed with bacteriological evidence in China [5]. In this study, we report human plague cases associated with Tibetan sheep on the Qinghai-Tibet plateau since the 1950s. Meanwhile, to further determine the ecological function of Tibetan sheep in *Y*. *pestis* endemic epidemics, we performed a genome-wide single nucleotide polymorphism (SNP) analysis of Tibetan sheep-related plague events, including pathogens isolated from humans, Tibetan sheep, and marmots. The genome-wide SNP analysis confirmed that the human plague strains were transmitted from Tibetan sheep, while the Tibetan sheep plague strains originated from marmots.

## Materials and methods

### Ethics statement

This study was approved by the Ethics Committee of the Qinghai Institute for Endemic Disease Control and Prevention (FLW2013-001) and the Institute for Communicable Disease Control and Prevention (ACUC2013-002). All animal plague surveillance procedures were performed in accordance with the National Regulations for the Administration of Affairs Concerning Experimental Animals approved by the State Council. All procedures were in accordance with the ethical standards of the National Research Committee.

### Isolation and identification of *Y*. *pestis*

**Fig 1:**
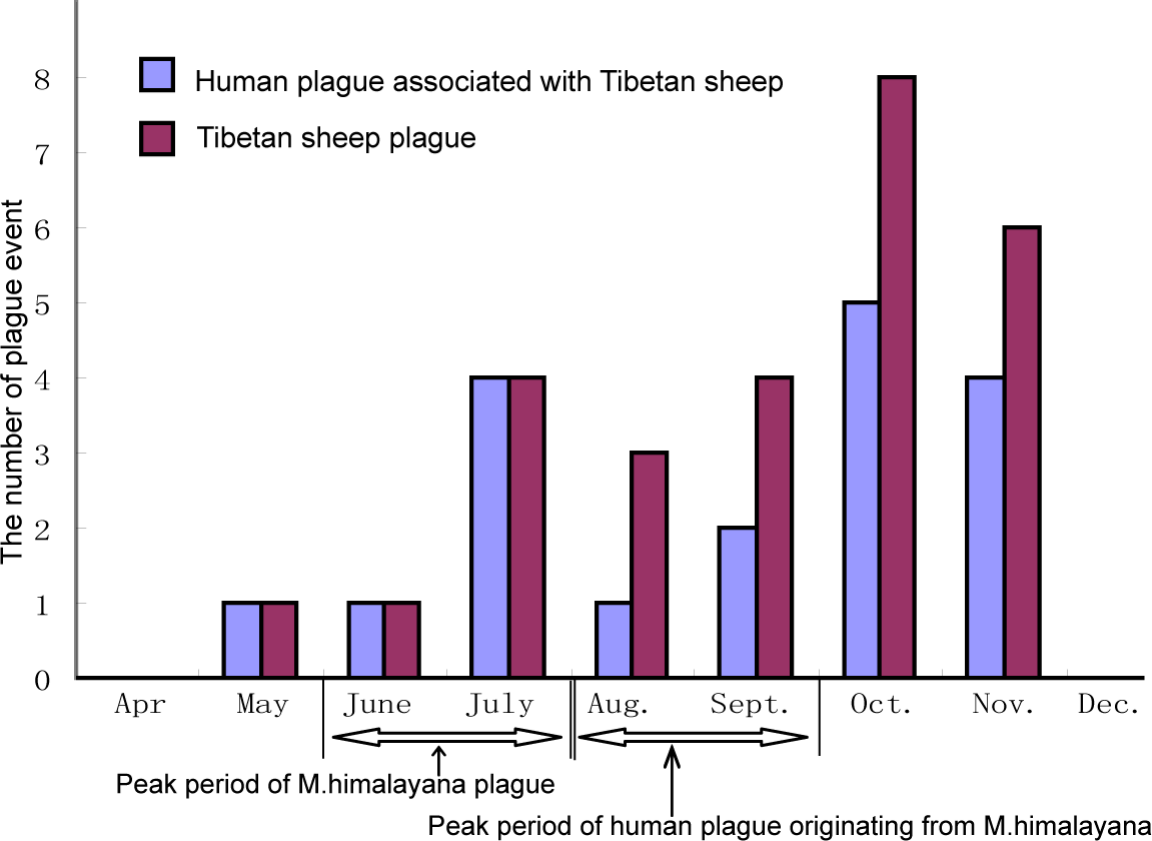
Times of occurrence of human plague events associated with Tibetan sheep and Tibetan sheep plagues on the Qinghai-Tibet plateau.

*Y*. *pestis* was isolated and identified by Gram staining, the reverse indirect hemagglutination assay, and the bacteriophage lysis test. All *Y*. *pestis* strains isolated from Tibetan sheep (15) or humans (7) associated with Tibetan sheep on the Qinghai-Tibet plateau were included (S1 Fig and S2 Table). The 18 outbreaks of human infection were designated from A to R (Fig 1 and S1 Table). The Tibetan sheep involved in human plague outbreaks based on epidemiological investigations were designated using the same alphabetic code (see S1 Table). In addition, 14 *Y*. *pestis* strains isolated from *M*. *himalayana* were selected; whenever possible, they were from the same region as the Tibetan sheep and in the same year in order to match the isolates from Tibetan sheep plague and human plague. Furthermore, two *Y*. *pestis* strains isolated from patients infected by *M*. *himalayana* in Nangqian County (2004) were also included [7]. All the strains were collected from the Qinghai Institute for Endemic Disease Control and Prevention, Xining, China. In addition, we plotted the geographical distribution of human plague, Tibetan sheep plague, and the isolates involved on a satellite map sourced from the Institute of Geographical Sciences and Natural Resources Research, Chinese Academy of Sciences, and we have received permission to publish it under a CC BY license from the institute.

### DNA preparation

A total of 38 *Y*. *pestis* strains isolated from Tibetan sheep or humans or *M*. *himalayana* were included in this study. Genomic DNA from each bacterium was extracted using the following method in a Biosafety Level 3 Laboratory of the Qinghai Institute for Endemic Disease Control and Prevention. *Y*. *pestis* strains were cultivated in Luria–Bertani broth at 28°C for 48 h, and the collected strains were suspended in 0.5 ml of TE buffer (10.0 mM Tris-HCl [pH 8], 1.0 mM EDTA) and incubated at 28°C for 20 min, Then 80 μl of 10% SDS was added to the preparation (10 μg in 1 ml PBS), and maintained at 65°C for 10 min. Next, 20 μl RNase (10 mg/ml) was added, and the solution incubated at 37°C for 2 h. Following the addition of 10 μl of proteinase K, the preparation was incubated at 37°C for 2 h. The DNA was extracted twice with equal volumes of phenol and once with an equal volume of chloroform. The DNA was precipitated by adding two volumes of absolute ethanol. The precipitated DNA was washed with 70% ethanol and re-suspended in TE buffer (pH 8.0).

### Genome sequencing and SNP analysis

The 38 isolates were sequenced using the Illumina HiSeq 2000 platform (Illumina, San Diego, CA). Two paired-end libraries were constructed with average insertion lengths of 500 bp and 3,000 bp. The raw data were filtered by FastQC, and then the clean data were assembled into contigs using SPAdes v3.9.1. Gene prediction was performed using Glimmer with the default parameters. The whole-genome raw SNPs were detected through pairwise comparisons of *Y*. *pestis* genomes to the reference genome of the Angola strain (NC_010159) [8] using Bowtie 2 software [9] and MUMmer [10] with the default parameters. Twenty-one completed genomes or draft genomes obtained from the NCBI database were also included in the analysis [1,11–17] (S2 Table). Then the SNPs were combined, and those of low quality (read depth <5) and those located within 5 bp on the same chromosome were removed to avoid the effect of recombination. A phylogenetic tree of *Y*. *pestis* was established based on these SNPs with the Bayesian evolutionary method in BEAST software [18] using the 38 *Y*. *pestis* genomes from our study and the 21 genome sequences of *Y*. *pestis* from GenBank and rooted with *Y*. *pseudotuberculosis* (IP32953) [1,13].

### Nucleotide sequence accession numbers

The sequencing data of the *Y*. *pestis* strains are available in GenBank under accession numbers SRP131404, and the genome sequences of 38 *Y*. *pestis* strains sequenced in our study have been deposited in GenBank with accession Nos SRR6512812 to SRR6512849.

## Results

### Human plague associated with Tibetan sheep on the Qinghai-Tibet plateau

According to the epidemic history of plague on the Qinghai-Tibet plateau and the annual national plague surveillance data in China, a total of 18 human outbreaks (events, designated A to R) associated with Tibetan sheep have occurred since 1956 (S1 Table and S1 Fig). Among these events, a total of 78 human cases associated with Tibetan sheep (cases of original infection and successive secondary generation) and 47 deaths were reported, of which 70 cases and 42 deaths occurred in Qinghai. In addition, 8 human cases (5 deaths) associated with Tibetan sheep occurred in Tibet. All index infectious cases had an exposure history of butchering or skinning diseased or dead Tibetan sheep. Massive deaths or larger numbers of infection cases mainly occurred in four events before 1975; for example in 1956, the index case (Tianjun county) suffered pneumonic plague and died after skinning a dead Tibetan sheep, and this individual infected a total of 13 individuals of whom 11 died. Eating meat from infected sheep that is not fully cooked is another cause of human plague infection, such as those in 1961 (Dulan county), 1963 (Yushu county), and 1965 (Zhaduo county), that caused 26 cases of infection due to eating the meat; only the index individual in each outbreak slaughtered or skinned a diseased or dead Tibetan sheep.

Considering the months in which Tibetan sheep plague, *M*. *himalayana* plague, and human plague events have occurred on the plateau since 1956, 14 of the 27 Tibetan sheep plague events occurred during October and November. In contrast, the peak occurrence of *M*. *himalayana* plague was during June and July and usually ended in October (National Plague Surveillance data and reference [5]). The plague in Tibetan sheep clearly lagged that in *M*. *himalayana* (Wilcoxon signed rank test, P <0.05). In addition, 9 of the 18 human plague events in which the index case(s) originated from Tibetan sheep occurred during October and November, while the peak months of human plague originating from *M*. *himalayana* were during August and September [5] (Fig 1). From 1997 to 2016, no human plague cases were caused by Tibetan sheep due to active prevention and intervention measures in Qinghai, even though *Y*. *pestis* was still isolated from local Tibetan sheep and Tibetan goats on the Qinghai-Tibet plateau.

### Identification of SNPs and the phylogenetic relationships of *Y*. *pestis*

The genomic sequences of the 38 isolates of *Y*. *pestis* were assembled *de novo*, producing 52 contigs and 70 scaffolds on average. The number of genes per strain ranged from 2,623 to 2,990. The phylogenetic tree of *Y*. *pestis* was established using all isolates in our study as well as 21 complete genomes or draft genome sequences from the NCBI GenBank database (S2 Table). We identified 1663 high-quality SNPs compared with the reference genomes and 216 within our isolates, with 39–63 SNPs per genome. Among these SNPs, 149 were located in 143 genes, including 41 synonymous SNPs and 108 nonsynonymous SNPs, with 1–2 in each gene, whereas the remaining 67 SNPs were located in intergenic regions. The 108 nonsynonymous sites were distributed among 106 genes.

**Fig 2:**
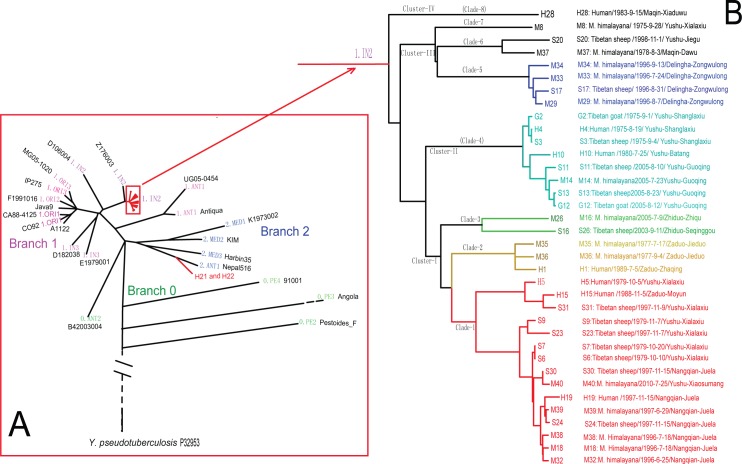
Phylogenetic tree of *Y*. *pestis* and the population structure of 38 *Y*. *pestis* isolates in our study. Phylogenetic relationships and population structure were determined by Bayesian evolutionary analysis based on genomic SNPs. Lower left (A): phylogenetic tree of *Y*. *pestis* yielded by genome-wide SNPs in 38 *Y*. *pestis* from our study and 21 genomic sequences of *Y*. *pestis* from GenBank, rooted in *Y*. *pseudotuberculosis* (IP32953). Black text: names of genomic sequences (S2 Table); colored text: branch and population names. Strains isolated from Tibetan sheep or associated human plague cases as well as a panel of isolates from *M*. *himalayana* are marked in red. Right (B): reconstruction of the population structure of 36 *Y*. *pestis* in the 1.IN2 lineage from the SNP assignments.

The phylogenetic relationships we constructed (Fig 2A) were very similar to the genomic maximum parsimony tree reported previously [1]. The nomenclature of the lineages in the phylogenetic tree are according to the literature [1,19]. The pathogens associated with Tibetan sheep plague were clustered into the 1.IN2 lineage in the phylogenetic tree. These strains were comparatively closer to *Y*. *pestis* Z176003, which was isolated from *M*. *himalayana* in Naqu County, Tibet, in 1976 [11]. In addition, strains H21 and H22 (human plague isolates originating from *M*. *himalayana* in Bangqian Village, Nangqian County in 2004) were clustered in 2.ANT1.

### Population structure of *Y*. *pestis* isolates associated with Tibetan sheep

*Y*. *pestis* isolated from Tibetan sheep or local *M*. *himalayana* all fermented glycerin and reduced nitrate to nitrite, i.e., they belonged to biovar antique, the same as human plague in this focus. Combining the epidemiological information (S1 Table and S2 Table) and the population structure based on the genome-wide SNP analysis, we divided the 36 *Y*. *pestis* in the 1.IN2 lineage (including those originating from Tibetan sheep (15) and humans (7) associated with Tibetan sheep, as well as 14 *Y*. *pestis* strains isolated from *M*. *himalayana*) into four clusters (I–IV), corresponding to eight clades (1–8) (Fig 2B). Generally, the clade-based classification agreed well with the geographical area, i.e., the strains isolated from the same area were found in the same clade (Fig 2B). In fact, where no geographic barrier existed between adjacent areas, the pathogens isolated from adjacent areas also grouped together; for example, Juela Village in Nangqian County and Xialaxiu Village in Yushu are adjacent, and the strains isolated from the two villages grouped into Clade-1; Shanglaxiu, Batang, and Guoqing Villages are neighbors, and the lineages were grouped in Clade-4. This shows that the genomic phylogenetic analysis of the Tibetan sheep-related strains have territory-specific characteristics.

In addition, in Clade-1 and Clade-4, the strains isolated in different years were grouped together. For example, the human plague cases and those corresponding to Tibetan sheep plague occurring in 1979 (in Xialaxiu Village, Yushu County) and in 1997 (in Juela Village, Nangqian County) were grouped together into Clade-1. In 1975, in Yushu County, the first human plague associated with Tibetan sheep was confirmed by bacteriological evidence. However, in 2005 in Yushu County, a larger-scale Tibetan sheep plague occurred, in which a total of 13 Tibetan sheep and 1 Tibetan goat in the same flock died. The isolates from these two events were grouped into Clade-4. This indicated that the same strains of *Y*. *pestis* successively caused Tibetan sheep or human plague outbreaks in these areas. Of course, some isolates could not be grouped together by event although the strains were isolated in the same area, such as lineages M8 and S20. In fact, finding any clear epidemiological connection between these two isolates and the rest was difficult. One possible explanation is the genomic diversity of the strains in these foci.

### Phylogenetic relationships among isolates from patients, Tibetan sheep, and marmots

In Clade-1, the *Y*. *pestis* isolated from patients (H19) and Tibetan sheep (S30 and S24) in Juela Village, Nangqian County in 1997, as well as isolates from *M*. *himalayana* (M39), were grouped together. According to the epidemiological information, the diseased herdsman (H19) suffered pneumonic plague after processing a dead Tibetan sheep (S24), and isolate S30 was obtained from a sheep in the same breeding herd as the dead sheep (S24). In addition, one strain (M39) from a dead *M*. *himalayana* in a sheep grazing area had been isolated four months earlier. In fact, a raging animal plague epidemic had occurred one year previously (in 1996) in Juela Village, and a total of three strains (M18, M32, and M38) were collected in the area (National Plague Surveillance data). The above isolates were grouped together in Clade-1. Two strains (S31 and S23) isolated from Tibetan sheep in Xialaxiu Village, Yushu County (adjacent to Nangqian County) also grouped into Clade-1. Furthermore, the strain (H5) from the human plague in 1979, the corresponding Tibetan sheep strains (S6 and S7), and some strains isolated from *M*. *himalayana* also clustered into Clade-1.

As noted above, in 2005, three *Y*. *pestis* isolates (S11, G12, and S13) were identified in two Tibetan sheep and one Tibetan goat from an outbreak of Tibetan sheep plague in Guoqing Village, Yushu. The *Y*. *pestis* isolated from the dead *M*. *himalayana* found in the same village and in the same year (2005) were clustered into the same clade (Clade-4). In fact, it was in Shanglaxiu Village, Yushu County, that the first human plague case associated with Tibetan sheep was confirmed in 1975. In addition, three *Y*. *pestis* strains isolated from dead patients and Tibetan sheep and Tibetan goats in the same herd were also clustered in Clade-4. Similar clustering of Tibetan sheep and *M*. *himalayana* was also found in Zongwulong Village, Delingha County, in 1996 (Clade-5). The above findings, together with the epidemiological connections, support the conclusion that human plague came from Tibetan sheep and Tibetan sheep plague originated from marmots.

## Discussion

### Human plague associated with Tibetan sheep plague in Qinghai

The Qinghai *M*. *himalayana* natural plague focus was first identified in 1954 as a result of the isolation of *Y*. *pestis* from a dead marmot in Qinghai Province [20]. *M*. *himalayana* is the primary plague host in this area [5]. According to plague surveillance data in Qinhai Province, a total of 468 human plague cases with 240 deaths were reported, of which 162 cases originated from *M*. *himalayana* (34.62%), 39 from Tibetan sheep (8.33%), 16 from carnivorous animals (3.42%), and 216 from successive infection of pneumonic plague by person-to-person transmission (46.15%)[5]. Tibetan sheep plague was sporadic on the Qinghai-Tibet plateau, and was restricted to areas that had *M*. *himalayana* plague epidemics. One previous investigation in Yushu Prefecture in 2005 found that the infection rate of *Y*. *pestis* in Tibetan sheep was 6.08% (64/1051) with serum titers in the range of 1:20 to 1:1280 [7]. Tibetan sheep-related human plague infection is always associated with slaughtering or skinning diseased or dead Tibetan sheep. Eating incompletely cooked meat from infected sheep or goats is another cause of human infection [5]. In previously research, the incidence of Tibetan sheep-related human plague outbreaks occurring in Qinghai Province between 1975–2009 were counted, and a total of 10 Tibetan sheep-related human plague outbreaks occurred during this period, resulting in 25 cases and 10 deaths, including bubonic plague (9), primary pneumonic plague (6), secondary pneumonic plague (6), septicemic plague (3), and intestinal plague(1) [2,3].

### Tibetan sheep infection from marmots

The even-toed ungulates (Artiodactyla), including camels and goats [21–24], donkeys, and cows [25], can be naturally infected by *Y*. *pestis*. Previous studies have shown that the sheep is a plague reservoir with high susceptibility and moderate sensitivity [26,27]. And, under natural circumstances, only individual Tibetan sheep in a flock are infected, and they do not become infected directly by sheep-to-sheep contact, even when the same flock contains a mixture of sick and healthy sheep [26]. These findings indicate that the ecological function of the Tibetan sheep in associated human plague should be considered as an intermediate or accidental host.

Another piece of supporting evidence is the fact that the occurrence of Tibetan sheep plague during the year lags behind the occurrence of *M*. *himalayana* plague. October and November were the high incidence months for the Tibetan sheep plague and human plague originated from Tibetan sheep. On the Qinghai-Tibet plateau, marmots begin hibernation from October to early November. One possible reason is that the fleas living in the caves escape after the marmots enter hibernation in October and attack other animals, such as Tibetan sheep. A minor peak for the human plague associated with Tibetan sheep occurs in June to July and presumably is caused by the massive death of marmots. Such an ecological change could also result in more fleas escaping from dead hosts and colonizing Tibetan sheep or human beings.

Several possible scenarios may explain how Tibetan sheep become infected by marmots. First, they could be infected by contact with the bodies of dead marmots. Our field observations showed that Tibetan sheep have a habit of licking the bodies of dead rodents such as marmots, which may be a means of ingesting micronutrients in the plateau environment. Previously, a study successfully induced plague infection by feeding or smearing *Y*. *pestis* in the mouths of Tibetan sheep [27]. Another possible cause is that Tibetan sheep could be infected by fleas such as *Callopsylla dolabris* or *Oropsylla silantiewi*. These are the main parasitic fleas in *M*. *himalayana*. Even though they have comparatively specific host selection, they have been found to attack human beings or other animals after the death of their preferred host [6]. Previous research has shown that *C*. *dolabris* and *O*. *silantiewi* bite and can suck the blood of Tibetan sheep in the laboratory, and the sheep can become infected and die after being challenged for 10 days [27]. The above evidence shows that fleas play an important role in *Y*. *pestis* transmission from marmots to Tibetan sheep.

Through genomic analysis, we confirmed that human plague came from Tibetan sheep, and Tibetan sheep plague originated from marmots. To the best of our knowledge, natural infection of sheep with *Y*. *pestis* is rare elsewhere in the world. The Tibetan sheep plague epizootic has some novel features, such as a complex transmission route, an extended epizootic period, and the possibility of transmission across long distances. Therefore, the hazards of Tibetan sheep plague should not be underestimated.

## Supporting information

S1 Fig Distribution of human plague associated with Tibetan sheep on the Qinghai-Tibet plateau. Yellow squares: 18 human plague outbreak events associated with sheep on the plateau since 1956. Numbers in brackets: number of human cases (including original infection and cases of successive secondary generation) followed by the number of deaths per event; red dots: areas of occurrence of human plague associated with sheep and the strains involved in this study. Lower left: location of the Qinghai-Tibet plateau; upper left: a Tibetan sheep (female). The satellite figure sourced from the Institute of Geographical Sciences and Natural Resources Research, Chinese Academy of Sciences, and we have received permission to publish this figure under a CC BY license from the institute.
(JPG)

S1 Table Human plague associated with Tibetan sheep on the Qinghai-Tibet plateau, 1956–2016.
(DOC)

S2 Table *Y*. *pestis* and corresponding animal or human plague outbreaks in this study. (DOC)

S3 Table SNPs detected in the sequenced *Y*. *pestis* strain. (XLSX)
