# Supplementary figures and images for: Human plague associated with Tibetan sheep originates in marmots
Source: PLoS Negl Trop Dis. 2018 Aug 16;12(8):e0006635. doi: 10.1371/journal.pntd.0006635 (PMC6095483; doi:10.1371/journal.pntd.0006635)

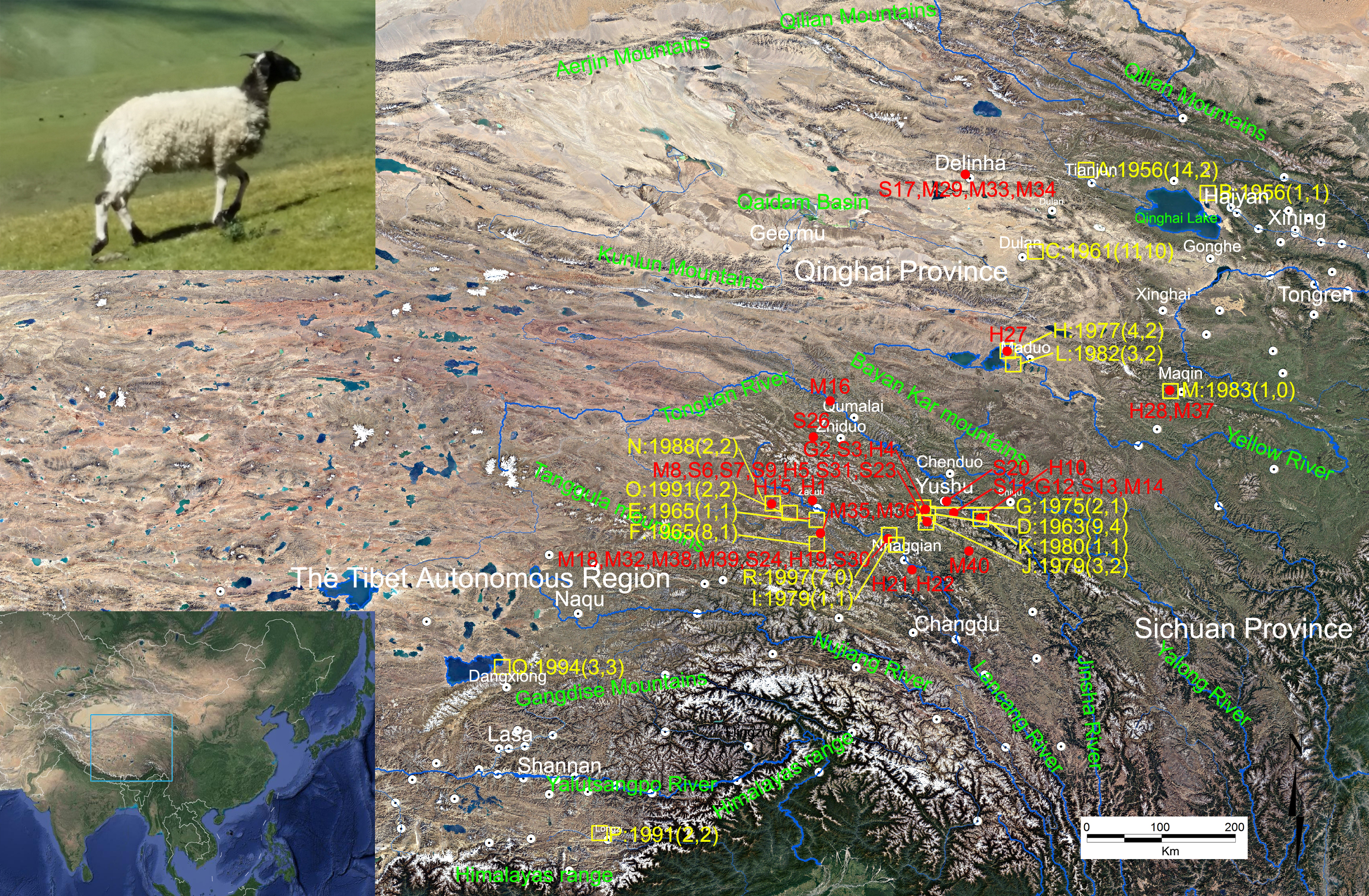

Supplement: S1 Fig — Yellow squares: 18 human plague outbreak events associated with sheep on the plateau since 1956. Numbers in brackets: number of human cases (including original infection and cases of successive secondary generation) followed by the number of deaths per event; red dots: areas of occurrence of human plague associated with sheep and the strains involved in this study. Lower left: location of the Qinghai-Tibet plateau; upper left: a Tibetan sheep (female). The satellite figure sourced from the Institute of Geographical Sciences and Natural Resources Research, Chinese Academy of Sciences, and we have received permission to publish this figure under a CC BY license from the institute. (JPG) [file pntd.0006635.s001.jpg]
