# Supplementary material for: Human plague associated with Tibetan sheep originates in marmots
Source: PLoS Negl Trop Dis. 2018 Aug 16;12(8):e0006635. doi: 10.1371/journal.pntd.0006635 (PMC6095483; doi:10.1371/journal.pntd.0006635)
Supplement: S1 Table — Human plague associated with Tibetan sheep on the Qinghai-Tibet plateau, 1956–2016. (DOC) [file pntd.0006635.s002.doc]

Supplemental Table 1. Human plague associated with Tibetan sheep and *Y. pestis* isolated from Tibetan sheep in Qinghai-Tibet Plateau（1956-2016）

| Date (Y-M-D) | Province (County/Village) | Route and number of human infections | Number of deaths | Strain source (number) | Code of human plague outbreak (strains isolated from human and Tibetan sheep ) | Strains from *M. himalayana* |
| --- | --- | --- | --- | --- | --- | --- |
| 1956-7-15 | Qinghai (Tianjun/Duoshike) | Skinning sheep (1)  Person-to-person (13) | 12 |  | A |  |
| 1956-10 | Qinghai (Haiyan/Reshui) | Skinning (1) | 1 |  | B |  |
| 1961- 10-15 | Qinghai (Dulan) | Skinning (1)  Eating meat (10) | 10 | Human (1) | C |  |
| 1963-9 | Qinghai (Yushu/Xialaxiu) | Skinning (1)  Eating meat (8) | 4 | Human (1) | D |  |
| 1965-5-4 | Qinghai (Zhaoduo/Angsai) | Skinning (1) | 1 | Human (1) | E |  |
| 1965-10-6 | Qinghai (Zhaduo/Jiezhaduo) | Skinning (1) Eating (8) | 1 | human (1) | F |  |
| 1966 to 1974 | None |  |  |  |  |  |
| 1975-8 | Qinghai (Yushu/Shanglaxiu) | Skinning (2) | 1 | Human (1) Tibetan sheep (1) Tibetan goat (1) | G (H4) (G2,S3) |  |
| 1975-9-28 | Qinghai (Yushu/Xialaxiu) |  |  |  |  | (M8） |
| 1977-11 | Qinghai (Maduo/Zhalinghu) | Skinning (4) | 2 |  | H |  |
| 1977-9-4 | Qinghai (Zaduo/Jiegu) |  |  |  |  | (M35, M36) |
| 1978-8-3 | Qinghai (Maqin/Dawu) |  |  |  |  | (M37) |
| 1979-6 | Qinghai (Nangqian/Nasuoni) | Skinning (1) | 1 |  | I |  |
| 1979-10-5 | Qinghai (Yushu/Xialaxiu) | Skinning (3) | 2 | Human (1) Tibetan sheep (2) | J (H5) (S6, S7, S9) |  |
| 1980-7 | Qinghai (Yushu/Batang) | Skinning (1) | 1 | Human (1) | K (H10) |  |
| 1982-7-4 | Qinghai (Maduo/Zhalinghu) | Skinning (2) | 2 | Human (1) | L (H27) |  |
| 1982-10 | Qinghai (Yushu/Xialaxiu) |  |  | *Procapra picticaudata* (1) |  |  |
| 1983-9-15 | Qinghai (Maqin/Xiadawu) | Skinning (1) | 0 | Human (1) | M (H28) |  |
| 1988-11-5 | Qinghai (Zhaduo/Moyun) | Skinning (2) | 2 | Human (1) | N (H15) |  |
| 1989-7-5 | Qinghai (Zhaduo/Zhaqing) | Skinning (2) | 2 | Human (1) | O (H1) |  |
| 1991-11-5 | Tibet (Longzi/Xinba) | Skinning (2) | 2 | Human (2) | P |  |
| 1992-10 | Tibet (Longzi) |  |  | Tibetan sheep (1) |  |  |
| 1994-10-15 | Tibet (Dangxiong/Namucuo) | Skinning (3) | 3 | Tibetan sheep (1) | Q |  |
| 1994-10 | Tibet (Biru) |  |  | Tibetan sheep (1) |  |  |
| 1996-9 | Tibet (Longzi/Liemai) |  |  | *Pseudois nayaur* (3) |  |  |
| 1996-8-31 | Qinghai (Delingha/Zongwulong) |  |  | Tibetan sheep (1) | (S17) | (M29, M33, M34) |
| 1997-11-15 | Qinghai (Nangqian/Juela) | Skinning (7) | 0 | Human (2), Tibetan sheep (2) | R (H19) (S30, S24) | (M18, M38, M39, M32) |
| 1997-11-7(9) | Qinghai (Yushu/Xialaxiu) |  |  | Tibetan sheep (2) | (S31,S23) |  |
| 1998-11-1 | Qinghai (Yushu/Jiegu) |  |  | Tibetan sheep (1) | (S20) |  |
| 2003-9-11 | Qinghai (Zhiduo/Seqinggou) |  |  | Tibetan sheep (1) | (S26) |  |
| 2005-8 | Qinghai (Yushu/Guoqing) |  |  | Tibetan goat (1) Tibetan sheep (2) | (S11, G12, S13) | (M14) |
| 2005-7-9 | Qinghai (Zhiduo/Zhiqu) |  |  |  |  | (M16) |
| 2010-7-25 | Qinghai (Yushu/Xiaosumang) |  |  |  |  | (M40) |
| 2005-2016 | None |  |  |  |  |  |
