# Supplementary material for: Human plague associated with Tibetan sheep originates in marmots
Source: PLoS Negl Trop Dis. 2018 Aug 16;12(8):e0006635. doi: 10.1371/journal.pntd.0006635 (PMC6095483; doi:10.1371/journal.pntd.0006635)
Supplement: S2 Table — (DOC) [file pntd.0006635.s003.doc]

Supplemental Table 2.

*Y. pestis* and corresponding animal or human plague outbreaks in this study

A: *Y. pestis* isolated from different hosts, including humans, Tibetan sheep, and *M. himalayana*.

| Strain code | Source | Date (Y-M-D) | Province (County/Village) | Human outbreak | Latitude longitude | Reference |
| --- | --- | --- | --- | --- | --- | --- |
| G2 | Tibetan goat | 1975-9-1 | Qinghai (Yushu/Shanglaxiu) | G | 96.58 N 32.91 E | In this study |
| S3 | Tibetan sheep | 1975-9-4 | Qinghai (Yushu/Shanglaxiu) | G | 96.58 N 32.85 E | In this study |
| H4 | Human | 1975-8-19 | Qinghai (Yushu/Shanglaxiu) | G | 96.51 N 32.89 E | In this study |
| H10 | Human | 1980-7-25 | Qinghai (Yushu/Batang) | K | 97.15 N 32.83 E | In this study |
| S11 | Tibetan sheep | 2005-8-10 | Qinghai (Yushu/Guoqing) |  | 96.80 N 33.03 E | In this study |
| G12 | Tibetan goat | 2005-8-12 | Qinghai (Yushu/Guoqing) |  | 96.80 N 33.03 E | In this study |
| S13 | Tibetan sheep | 2005-8-23 | Qinghai (Yushu/Guoqing) |  | 96.80 N 33.03 E | In this study |
| M14 | *M. himalayana* | 2005-7-23 | Qinghai (Yushu/Guoqing) |  | 96.80 N 33.03 E | In this study |
| S20 | Tibetan sheep | 1998-11-1 | Qinghai (Yushu/Jiegu) |  | 97.01 N 32.99 E | In this study |
| S17 | Tibetan sheep | 1996-8-31 | Qinghai (Delingha/Zongwulong) |  | 97.34 N 37.38 E | In this study |
| M29 | *M. himalayana* | 1996-8-7 | Qinghai (Delingha/Zongwulong) |  | 97.37 N 37.38 E | In this study |
| M8 | *M. himalayana* | 1975-9-28 | Qinghai (Yushu/Xialaxiu) |  | 96.62 N 32.65 E | In this study |
| M33 | *M. himalayana* | 1996-7-24 | Qinghai (Delingha/Zongwulong) |  | 97.34 N 37.38 E | In this study |
| M37 | *M. himalayana* | 1978-8-3 | Qinghai (Maqin/Dawu) |  | 100.23 N 34.48 E | In this study |
| M34 | *M. himalayana* | 1996-9-13 | Qinghai (Delingha/Zongwulong) |  | 97.34 N 37.38 E | In this study |
| H28 | Human | 1983-9-15 | Qinghai (Maqin/Xiadawu) | M | 100.21 N 34.49 E | In this study |
| S26 | Tibetan sheep | 2003-9-11 | Qinghai (Zhiduo/Seqinggou) |  | 95.33 N 33.89 E | In this study |
| M16 | *M. himalayana* | 2005-7-9 | Qinghai (Zhiduo/Zhiqu) |  | 95.49 N 34.30 E | In this study |
| M35 | *M. himalayana* | 1977-7-17 | Qinghai (Zaduo/Jieduo) |  | 95.30 N 32.60 E | In this study |
| M36 | *M. himalayana* | 1977-9-4 | Qinghai (Zaduo/Jieduo) |  | 95.30 N 32.60 E | In this study |
| H1 | Human | 1989-7-5 | Qinghai (Zaduo/Zhaqing) | O | 95.17 N 33.06 E | In this study |
| M18 | *M. himalayana* | 1996-7-18 | Qinghai (Nangqian/Juela) |  | 96.15 N 32.56 E | In this study |
| M38 | *M. himalayana* | 1996-7-18 | Qinghai (Nangqian/Juela) |  | 96.15 N 32.56 E | In this study |
| M39 | *M. himalayana* | 1997-6-29 | Qinghai (Nangqian/Juela) |  | 96.16 N 32.56 E | In this study |
| M32 | *M. himalayana* | 1996-6-25 | Qinghai (Nangqian/Juela) |  | 96.14 N 32.56 E | In this study |
| S24 | Tibetan sheep | 1997-11-15 | Qinghai (Nangqian/Juela) | R | 96.15 N 32.56 E | In this study |
| M40 | *M. himalayana* | 2010-7-25 | Qinghai (Yushu/Xiaosumang) |  | 97.25 N 32.35 E | In this study |
| H19 | Human | 1997-11-15 | Qinghai (Nangqian/Juela) | R | 96.14 N 32.55 E | In this study |
| S6 | Tibetan sheep | 1979-10-10 | Qinghai (Yushu/Xialaxiu) | J | 96.60 N 32.69 E | In this study |
| S7 | Tibetan sheep | 1979-10-20 | Qinghai (Yushu/Xialaxiu) | J | 96.61 N 32.65 E | In this study |
| S9 | Tibetan sheep | 1979-11-7 | Qinghai (Yushu/Xialaxiu) | J | 96.60 N 32.66 E | In this study |
| H5 | Human | 1979-10-5 | Qinghai (Yushu/Xialaxiu) | J | 96.62 N 32.66 E | In this study |
| H15 | Human | 1988-11-5 | Qinghai (Zaduo/Moyun) | N | 94.26 N 33.17 E | In this study |
| S31 | Tibetan sheep | 1997-11-9 | Qinghai (Yushu/Xialaxiu) | R | 96.59 N 32.65 E | In this study |
| S23 | Tibetan sheep | 1997-11-7 | Qinghai (Yushu/Xialaxiu) | R | 96.60 N 32.65 E | In this study |
| S30 | Tibetan sheep | 1997-11-15 | Qinghai (Nangqian/Juela) | R | 96.13 N 32.55 E | In this study |
| H21 | Human | 2004-10-11 | Qinghai (Nangqian/Naiyang) |  | 96.52 N 32.06 E | In this study |
| H22 | Human | 2004-10-15 | Qinghai (Nangqian/Naiyang) |  | 96.52 N 32.07 E | In this study |

B: Twenty-one *Y. pestis* with completed genomes or draft genomes obtained from the NCBI database

|  | Strain | **Biovar** | **Country** | Year | **Group** | **Accession Number** | Reference |
| --- | --- | --- | --- | --- | --- | --- | --- |
| 1 | Y.p. 91001 | Microtus | China | 1970 | 0.PE4 | NC_005810 | 13 |
| 2 | Y.p CO92 | Orientalis | USA | 1992 | 1.ORI1 | NC_003143 | 12 |
| 3 | Y.p Nepal516 | Antiqua | Nepal | 1967? | 2.ANT1 | NZ_ACNQ00000000 | 14 |
| 4 | Y.p Antiqua | Antiqua | Congo | 1965 | 1.ANT1 | NC_008150 | 14 |
| 5 | Y.p Pestoides F | Pestoides | USSR | <1984 | 0.PE2 | NC_009381 | 16 |
| 6 | Z176003 | Antiqua | China | 1976 | 1.IN2 | CP001593 | 11 |
| 7 | D106004 | Antiqua | China | 2006 | 1.IN2 | CP001585 | 11 |
| 8 | MG05-1020 | Orientalis | Madagascar | 2005 | 1.ORI3 | NZ_AAYS00000000 | 1 |
| 9 | IP275 | Orientalis | Madagascar | 1995 | 1.ORI3 | NZ_AAOS00000000 | 1 |
| 10 | F1991016 | Orientalis | China | 1991 | 1.ORI2 | NZ_ABAT00000000 | 13 |
| 11 | Java9 | Orientalis | Indonesia |  |  | [CP009996.1](https://www.ncbi.nlm.nih.gov/nuccore/CP009996.1) | 17 |
| 12 | CA88-4125 | Orientalis | USA | 1988 | 1.ORI1 | NZ_ABCD00000000 |  |
| 13 | A1122 | Orientalis | USA | 1939 |  | NC_017168 | 17 |
| 14 | D182038 | Antiqua | China | 1982 | 1.IN3 | CP001589 | 11 |
| 15 | E1979001 | Antiqua | China | 1979 | 1.IN3 | NZ_AAYV00000000 | 13 |
| 16 | UG05-0454 | Antiqua | Uganda | 2004 | 1.ANT1 | NZ_AAYR00000000 | 1 |
| 17 | K1973002 | Medievalis | China | 1973 | 2.MED2 | NZ_AAYT00000000 | 13 |
| 18 | KIM | Medievalis | Kurdistan | 1968 | 2.MED1 | NC_004088 | 15 |
| 19 | Harbin35 | Medievalis | China |  | 2.MED3 | [CP009704.1](https://www.ncbi.nlm.nih.gov/nuccore/CP009704.1) | 17 |
| 20 | B42003004 | Antiqua | China | 2003 | 0.ANT2 | NZ_AAYU00000000 | 13 |
| 21 | Angola | Pestoides | possibly Angola | <1984 | 0.PE3 | NC_010159 | 8 |
| 22 | IP32953 | Y. pseudotuberculosis | France |  | outgroup | NC_006155 | 1 |
